# Impact of Five Succinate Dehydrogenase Inhibitors on DON Biosynthesis of *Fusarium asiaticum*, Causing Fusarium Head Blight in Wheat

**DOI:** 10.3390/toxins11050272

**Published:** 2019-05-15

**Authors:** Chao Xu, Meixia Li, Zehua Zhou, Jiaosheng Li, Dongming Chen, Yabing Duan, Mingguo Zhou

**Affiliations:** 1 College of Plant Protection, Nanjing Agricultural University, Nanjing 210095, China; 2016102130@njau.edu.cn (C.X.); 2016102106@njau.edu.cn (M.L.); 2014202047@njau.edu.cn (Z.Z.); 2016102114@njau.edu.cn (J.L.); 2016102131@njau.edu.cn (D.C.); 2 State & Local Joint Engineering Research Center of Green Pesticide Invention and Application, Nanjing 210095, China

**Keywords:** deoxynivalenol, *Fusarium asiaticum*, succinate dehydrogenase inhibitor, glycolysis, tricarboxylic acid cycle, toxisomes

## Abstract

Deoxynivalenol (DON) is a class of mycotoxin produced in cereal crops infected with *Fusarium graminearum* species complex (FGSC). In China, FGSC mainly includes *Fusarium asiaticum* and *F. graminearum*. DON belongs to the trichothecenes and poses a serious threat to the safety and health of humans and animals. Succinate dehydrogenase inhibitors (SDHIs) are a class of fungicides that act on succinate dehydrogenase and inhibit the respiration of pathogenic fungi. In this study, the fungicidal activities of five SDHIs, including fluopyram, flutolanil, boscalid, benzovindiflupyr, and fluxapyroxad, against FGSC were determined based on mycelial growth and spore germination inhibition methods. The five SDHIs exhibited better inhibitory activities in spore germination than mycelial growth. Fluopyram exhibited a higher inhibitory effect in mycelial growth and spore germination in comparison to the other four SDHIs. In addition, the biological characteristics of *F. asiaticum* as affected by the five SDHIs were determined. We found that these five SDHIs decreased DON, pyruvic acid and acetyl-CoA production, isocitrate dehydrogenase mitochondrial (ICDHm) and SDH activities, and NADH and ATP content of *F. asiaticum* but increased the citric acid content. In addition, *TRI5* gene expression was inhibited, and the formation of toxisomes was disrupted by the five SDHIs, further confirming that SDHIs can decrease DON biosynthesis of *F. asiaticum*. Thus, we concluded that SDHIs may decrease DON biosynthesis of *F. asiaticum* by inhibiting glycolysis and the tricarboxylic acid (TCA) cycle. Overall, the findings from the study will provide important references for managing Fusarium head blight (FHB) caused by FGSC and reducing DON contamination in *F. asiaticum*-infected wheat grains.

## 1. Introduction

Fusarium head blight (FHB) is a devastating disease in wheat and other small grain cereals and is caused by *Fusarium graminearum* species complex (FGSC), including at least 16 distinct and cryptic species, and some species have specific geographical distributions [1,2]. In China, FGSC causing FHB is mainly composed of *F. asiaticum* and *F. graminearum* [3,4]. The disease can not only cause serious yield and quality losses in many wheat-growing regions, but also FHB pathogens can produce a series of trichothecene mycotoxins in FGSC-infected wheat grains, including deoxynivalenol (DON) and its acetylated derivatives (3AcDON, 15AcDON) and nivalenol (NIV), thus posing a grave threat to the safety and health of humans and animals [5]. DON has been demonstrated to be the most common contamination associated with FGSC-infected wheat grains and can cause hematic and anorexic syndromes as well as neurotoxic and immunotoxic effects in mammals. Additionally, DON has also been reported as an important virulence factor of FGSC [6,7].

The control of FHB always depends on chemical fungicides. Previous studies have shown that the resistance of benzimidazole is already widespread in China, especially in eastern China, and that there is a high resistance risk of FGSC to phenamacril [8,9,10]. Accordingly, it is of great importance to discover and develop novel fungicides that exhibit inhibitory effects on the fungal growth and DON biosynthesis of FHB pathogens. Succinate dehydrogenase inhibitors (SDHIs) are a new class of chemical fungicides. Previous studies have demonstrated that SDHIs target enzyme complex II of the mitochondrial respiratory electron transport chain, namely succinate dehydrogenase (SDH) or succinate quinone reductase (SQR) in phytopathogenic fungi [11,12]. The enzyme complex II is also an important functional part of the tricarboxylic acid (TCA) cycle and is linked to mitochondrial respiratory electron transport chain for catalysis of the coupling reaction from succinic acid oxidation to fumaric acid and reduction from ubiquinone to ubiquinol. It includes four subunits: Flavoprotein (SdhA), iron-sulfur protein (SdhB), and two other integral membrane proteins (SdhC and SdhD) [13,14]. In terms of chemical structure, SDHIs contain an amide group (-CONH-). Most of the newly developed fungicides are based on the original reactive group as a backbone. At present, SDHIs have been widely applied for controlling many plant diseases [15,16,17,18]. However, SDHIs are rarely used to control FHB, especially in the control of DON production in wheat grains. In this study, the effects of five SDHIs, fluopyram, flutolanil, boscalid, benzovindiflupyr, and fluxapyroxad, in inhibiting mycelial growth, spore germination of FGSC, and DON biosynthesis of *F. asiaticum* were determined. This study also evaluated the expression of *TRI5* gene, which is the DON biosynthesis-associated gene. In addition, the impacts of five SDHIs on DON biosynthesis-associated biological characteristics such as pyruvic acid, acetyl-CoA, ATP, citric acid and activities of several key enzymes were evaluated in vitro. Finally, the effect of these five SDHIs on toxisomes was investigated using a confocal laser scanning microscope.

## 2. Results

### 2.1. Sensitivity of FGSC to Five Succinate Dehydrogenase Inhibitors

In this study, the sensitivity tests of 13 FGSC strains to five SDHIs were performed based on mycelial growth and spore germination inhibition methods. For mycelial growth, the EC_50_ values of 13 FGSC strains to fluopyram ranged from 1.65 to 10.0 μg/mL (Table 1). Additionally, the EC_50_ values of 13 FGSC strains to flutolanil, boscalid, benzovindiflupyr, and fluxapyroxad were higher than 100 μg/mL. This suggested that fluopyram exhibits a better inhibitory effect in mycelial growth of FGSC compared to the other four SDHIs. For spore germination, the EC_50_ values ranged from 2.32 to 4.24 μg/mL for flutolanil, 1.19 to 3.06 μg/mL for boscalid, 1.79 to 2.98 μg/mL for benzovindiflupyr, 2.08 to 3.99 μg/mL for fluxapyroxad, and 0.39 to 0.74 μg/mL for fluopyram, respectively (Table 2). Fluopyram also exhibited a better inhibitory activity in spore germination than the other four SDHIs. The results suggested that the five SDHIs exhibited a better inhibitory effect on spore germination than mycelial growth of FGSC.

### 2.2. DON Production and TRI5 Gene Expression

**Figure 1:**
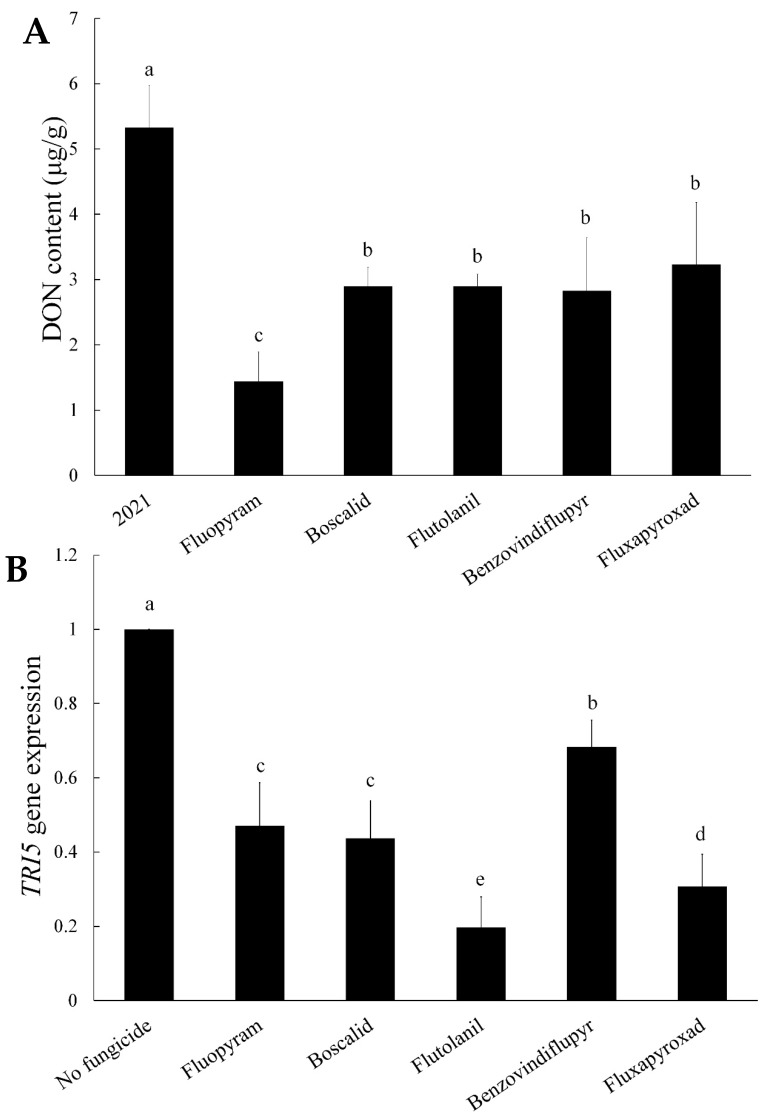
Effects of the five SDHIs in inhibiting deoxynivalenol (DON) production and *TRI5* gene expression in *F. asiaticum* in vitro. (**A**) The amount of DON produced by the wild-type strain 2021 in GYEP as affected by the five SDHIs. (**B**) The relative expression of the *TRI5* gene in the wild-type strain 2021 in GYEP as affected by the five SDHIs. The final concentrations were 0.54 μg/mL for fluopyram, 2.96 μg/mL for flutolanil, 1.52 μg/mL for boscalid, 1.79 μg/mL for benzovindiflupyr, and 3.16 μg/mL for fluxapyroxad, respectively. Values are means and standard errors of three replicates. Means with different letters are significantly different (*p* < 0.05, ANOVA, LSD).

To explore the inhibitory effect of SDHIs in DON biosynthesis, DON production and *TRI5* gene expression of *F. asiaticum* strain 2021 treated with SDHIs were determined. All fungicide treatments significantly decreased the DON content. Under treatments of flutolanil, boscalid, fluxapyroxad, and benzovindiflupyr, the DON content of 2021 decreased 40–50%, but decreased by approximately 70% under treatment of fluopyram (Figure 1A). 

The *TRI5* gene is a key gene for DON biosynthesis [19]. The transcriptional level of the *TRI5* gene in the strains was assayed by qRT-PCR. The *TRI5* gene was significantly down-regulated in the strain 2021 when treated with these five SDHIs (Figure 1B). Therefore, *TRI5* expression indicated that SDHIs, especially fluopyram, can significantly reduce the biosynthesis of DON in *F. asiaticum*.

### 2.3. Pyruvic Acid and Acetyl-CoA Content

**Figure 2:**
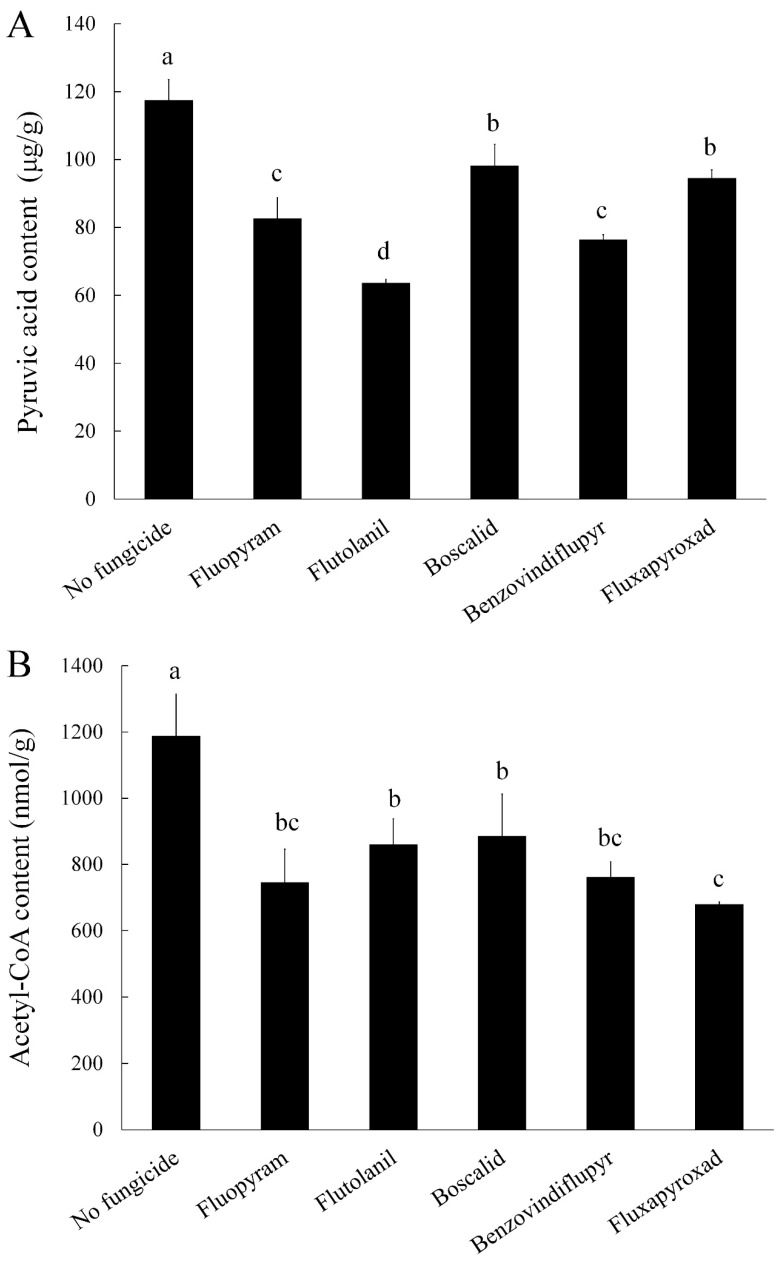
Impacts of the five SDHIs on pyruvic acid (**A**) and acetyl-CoA (**B**) production in the wild-type strain 2021. The final concentrations were 0.54 μg/mL for fluopyram, 2.96 μg/mL for flutolanil, 1.52 μg/mL for boscalid, 1.79 μg/mL for benzovindiflupyr, and 3.16 μg/mL for fluxapyroxad, respectively. Values are means and standard errors of three replicates. Means with different letters are significantly different (*p* < 0.05, ANOVA, LSD).

Pyruvic acid and acetyl-CoA are the main precursors for DON biosynthesis [20,21]. The above described results indicated that SDHIs can effectively inhibit DON biosynthesis. To further reveal the relation between DON biosynthesis and pyruvic acid or acetyl-CoA, we determined the pyruvic acid and acetyl-CoA content under the same culture conditions as DON determination. The results showed that the pyruvic acid and acetyl-CoA content of the strain 2021 significantly decreased when treated with these five SDHIs (Figure 2). We concluded that SDHIs inhibit the DON biosynthesis of *F. asiaticum* by decreasing pyruvic acid and acetyl-CoA production.

### 2.4. The Relative Expression of Key Genes in the Glycolysis Pathway

**Figure 3:**
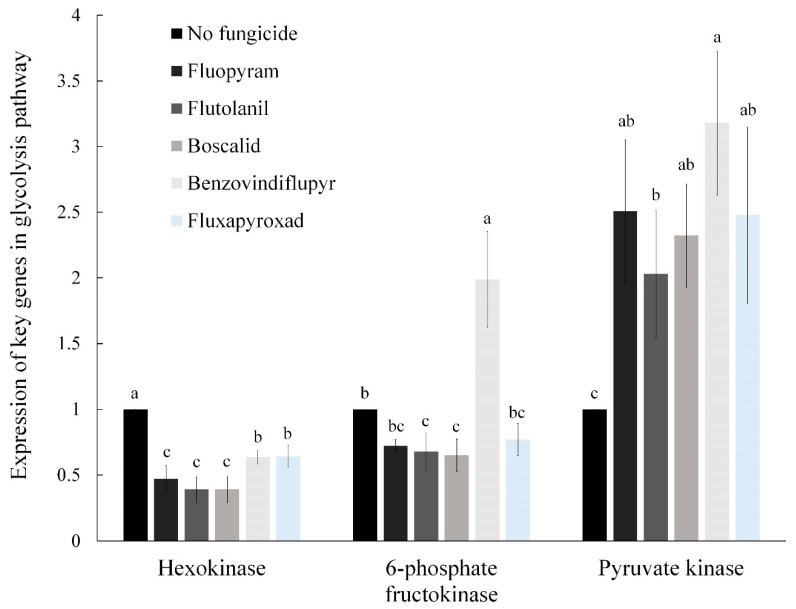
Relative expression levels of three key genes (hexokinase, 6-phosphate fructokinase, and pyruvate kinase) in the glycolysis pathway of the wild-type strain 2021 as affected by the five SDHIs. The final concentrations were 0.54 μg/mL for fluopyram, 2.96 μg/mL for flutolanil, 1.52 μg/mL for boscalid, 1.79 μg/mL for benzovindiflupyr, and 3.16 μg/mL for fluxapyroxad, respectively. Values are means and standard errors of three replicates. Means with different letters are significantly different (*p* < 0.05, ANOVA, LSD).

Pyruvic acid is mainly derived from the glycolysis pathway. We found that SDHIs can significantly inhibit the pyruvic acid content. Here, we determined the relative expression levels of three key genes hexokinase (FGSG_00500), 6-phosphate fructokinase (FGSG_09456), and pyruvate kinase (FGSG_07528) in the glycolysis pathway. We found that fluopyram, flutolanil, boscalid, and fluxapyroxad caused down-regulated expression of hexokinase and 6-phosphate fructokinase but up-regulated the expression of pyruvate kinase. Strangely, the relative expression of 6-phosphate fructokinase increased when treated with benzovindiflupyr, and the changes in the relative expression of the other two genes were consistent with the above treatment (Figure 3). Hexokinase and 6-phosphate fructokinase are the rate-limiting enzymes in the glycolysis pathway. The results demonstrate that these five SDHIs fungicides can effectively inhibit the glycolysis pathway of *F. asiaticum*.

### 2.5. Citric Acid Content

**Figure 4:**
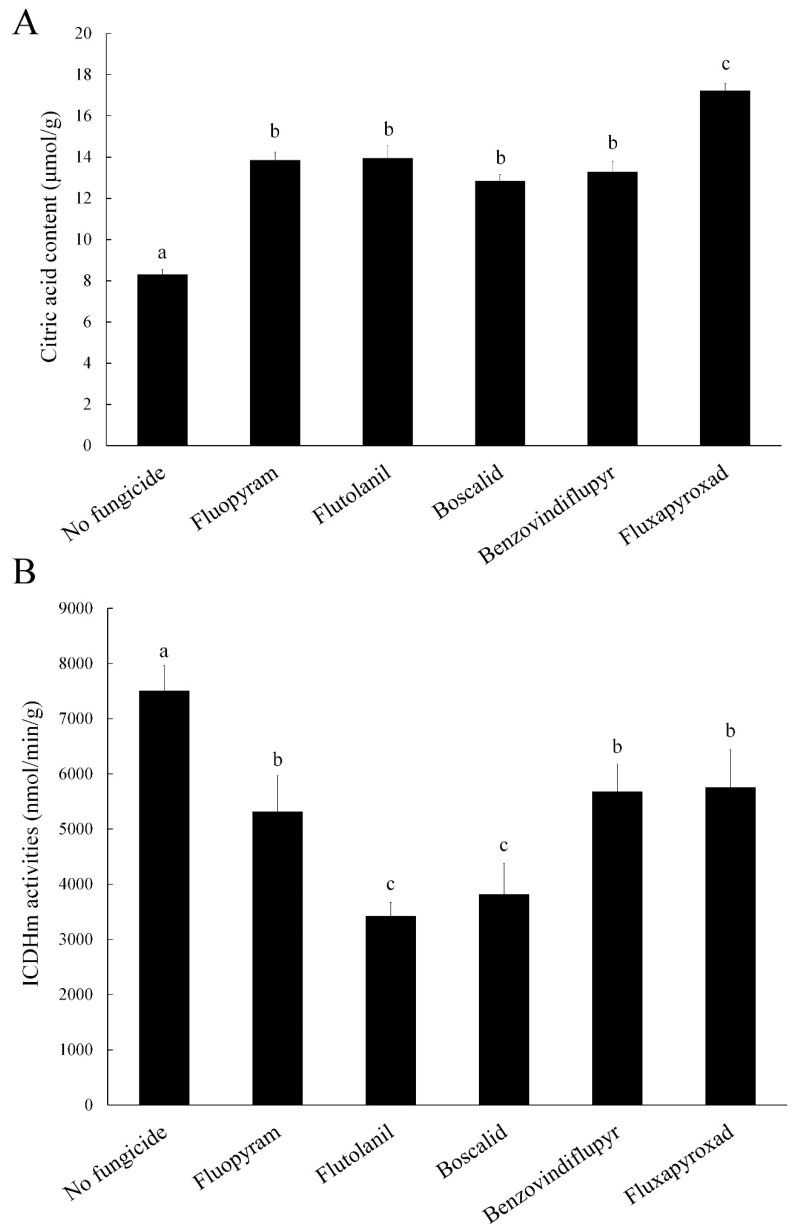
Impacts of the five SDHIs on citric acid content (**A**) and Isocitrate Dehydrogenase Mitochondrial (ICDHm) activities (**B**) in the wild-type strain 2021. The final concentrations were 0.54 μg/mL for fluopyram, 2.96 μg/mL for flutolanil, 1.52 μg/mL for boscalid, 1.79 μg/mL for benzovindiflupyr, and 3.16 μg/mL for fluxapyroxad, respectively. Values are means and standard errors of three replicates. Means with different letters are significantly different (*p* < 0.05, ANOVA, LSD).

Citric acid is the first product of the TCA cycle, and previous studies have shown that its accumulation inhibits glycolysis [22,23]. Therefore, we determined the content of citric acid of the strain 2021 after treatment. The results indicated that citric acid strongly increased in *F. asiaticum* strain 2021 treated with these five SDHIs (Figure 4A).

### 2.6. Isocitrate Dehydrogenase Mitochondrial (ICDHm) Activity

ICDHm is the rate-limiting enzyme in the TCA cycle [24,25], and we are curious about the effects on the rate of the TCA cycle reaction under treatment of these five SDHIs. One unit of enzyme activity is defined as the production of 1 nmol of NADH per gram of mycelium per minute. After treatment, the ICDHm activities of the strain 2021 were all significantly lower than that of the control (Figure 4B). These results indicate that SDHIs can effectively inhibit the rate of the TCA cycle of *F. asiaticum*.

### 2.7. Succinate Dehydrogenase (SDH) and NADH Dehydrogenase Activities

**Figure 5:**
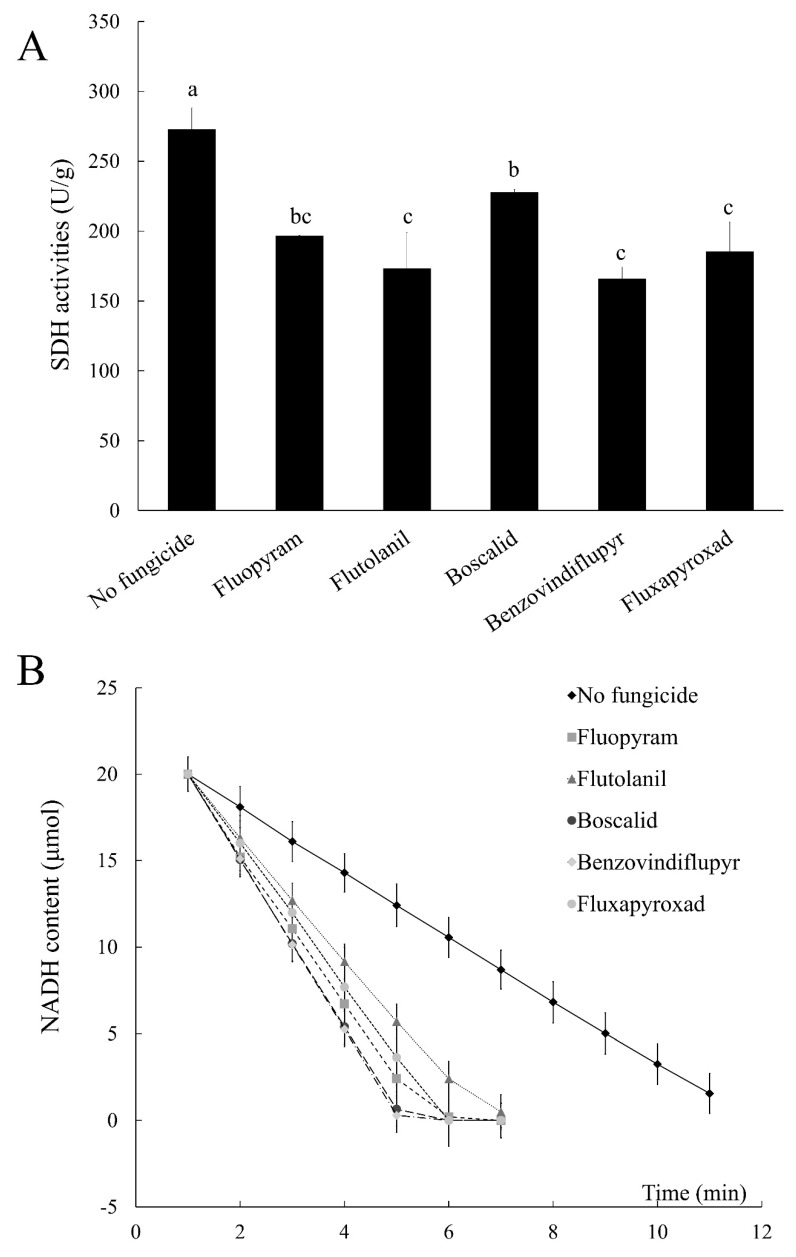
Impacts of the five SDHIs on SDH activities (**A**) and the rate of NADH decomposition (**B**) in the wild-type strain 2021. NADH dehydrogenase activity was defined as the rate of decomposition of NADH. The faster the rate of decomposing NADH, the higher the activity of NADH dehydrogenase. The final concentrations were 0.54 μg/mL for fluopyram, 2.96 μg/mL for flutolanil, 1.52 μg/mL for boscalid, 1.79 μg/mL for benzovindiflupyr, and 3.16 μg/mL for fluxapyroxad, respectively. Values are means and standard errors of three replicates. Means with different letters are significantly different (*p* < 0.05, ANOVA, LSD).

NADH dehydrogenase is an upstream complex of SDH, and we wondered what changes in NADH dehydrogenase would occur after inhibiting SDH activity. One unit of succinate dehydrogenase activity is defined as 1 nmol of 2,6-dichlorophenol indophenol per gram of tissue per minute. One unit of NADH dehydrogenase activity is defined as the rate of decomposition of NADH. Treated with these five SDHIs, the succinate dehydrogenase activity of 2021 was significantly lower than that of the control (Figure 5A). However, the NADH dehydrogenase activity of 2021 was significantly higher than that of the control (Figure 5B). NADH dehydrogenase plays a role of catalyzing the transfer of two electrons from NADH to coenzyme Q in the mitochondrial electron transport chain. The function of succinate dehydrogenase is to catalyze the transfer of electrons from succinic acid to FAD and iron-sulfur protein to coenzyme Q [14]. We surmised that after the treatment, the activity of succinate dehydrogenase is inhibited, and the transfer of electrons from succinic acid to coenzyme Q is reduced. The strains are compensated for the loss of such electron transport, and thus there is an increase in the number of electrons transferred from NADH to coenzyme Q. The rate of consumption of NADH is accelerated, and the activity of NADH dehydrogenase is enhanced.

### 2.8. ATP Content

**Figure 6:**
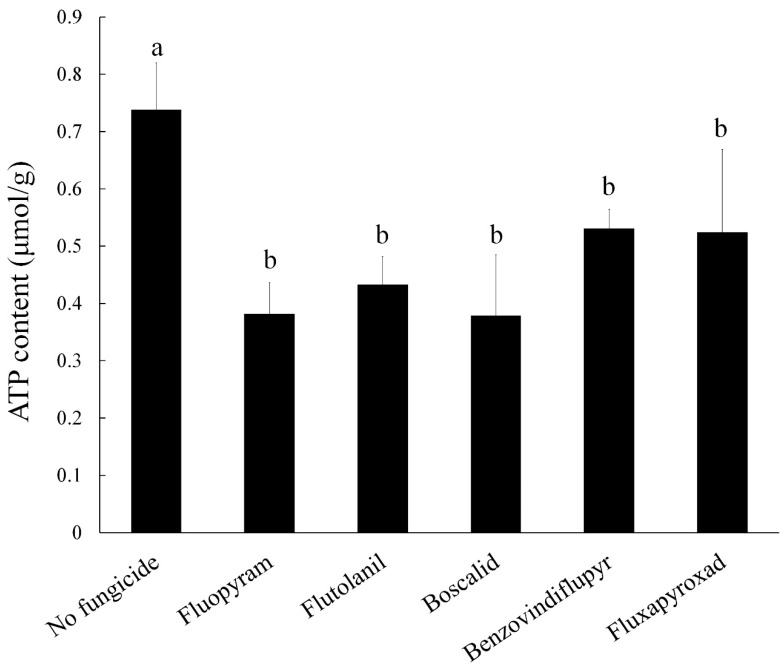
Impacts of the five SDHIs on ATP content in the wild-type strain 2021. The final concentrations were 0.54 μg/mL for fluopyram, 2.96 μg/mL for flutolanil, 1.52 μg/mL for boscalid, 1.79 μg/mL for benzovindiflupyr, and 3.16 μg/mL for fluxapyroxad, respectively. Values are means and standard errors of three replicates. Means with different letters are significantly different (*p* < 0.05, ANOVA, LSD).

SDHI fungicides are respiratory inhibitors that effectively inhibit the synthesis of energy in the strain, so we measured the ATP production in 2021 after treatment. It is unclear whether the content of ATP has an effect on the biosynthesis of DON. The quantification data indicated that ATP production in 2021 decreased after treatment compared to the control (Figure 6). The results showed that the energy metabolism in *F. asiaticum* can be affected when treated with these five SDHIs.

### 2.9. Impact on Toxisomes

**Figure 7:**
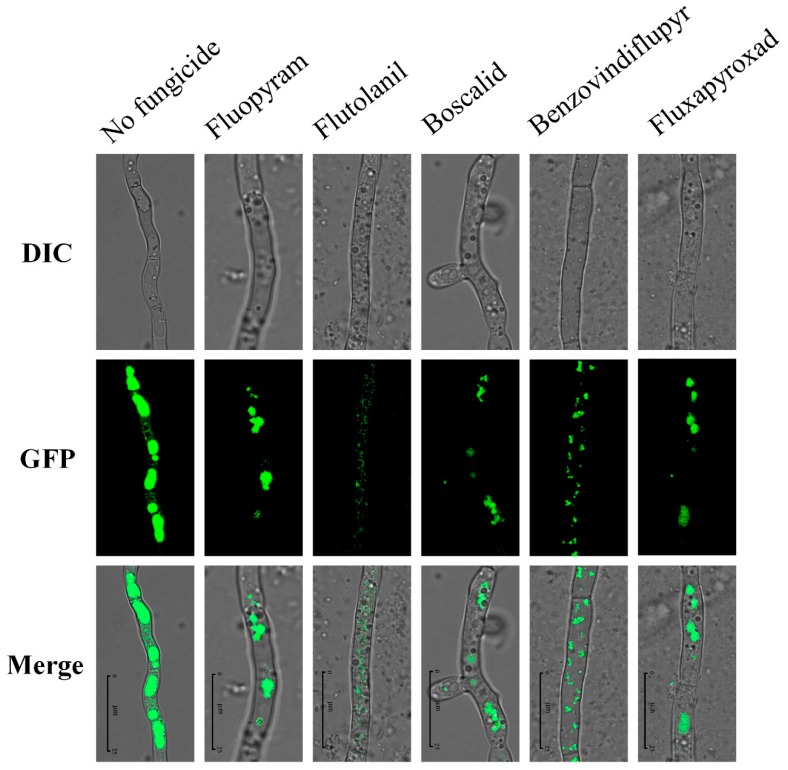
The morphology of the toxisomes in the wild-type strain 2021 as affected by the five SDHIs. The final concentrations were 0.54 μg/mL for fluopyram, 2.96 μg/mL for flutolanil, 1.52 μg/mL for boscalid, 1.79 μg/mL for benzovindiflupyr, and 3.16 μg/mL for fluxapyroxad, respectively. Bar = 25 μm. DIC: Differential interference contrast; GFP: Green fluorescence protein; Merge: Combination of DIC and GFP images. The pictures were captured under a ×100 oil objective.

Toxisomes are special spherical structures formed by *F. graminearum* under the conditions of toxin production. A previous study showed that the *TRI1* gene was localized to the toxisomes [26]. To further investigate the impact of five SDHIs in inhibiting the DON production of *F. asiaticum*, we labeled the GFP green fluorescent marker on the *TRI1* gene in 2021 to observe the effect of the five SDHIs on the morphology of the toxisomes. After the 2021 labeled with *TRI1*-GFP was cultured for 3 days in GYEP, we successfully observed a similar toxisome structure. Under the treatment of these five SDHIs, we found that the structure of the toxisomes was destroyed and the fluorescence intensity was weakened (Figure 7). The results indicated that SDHIs can destroy the structure of toxisomes, causing a reduction in the DON biosynthesis of *F. asiaticum*.

## 3. Discussion

FHB caused by FGSC is an economically important fungal disease on various cereals [27,28]. In addition to the loss of yield, the mycotoxins produced by FGSC in infected cereals pose a grave threat to the safety and health of humans and animals [4,5,29]. Since most wheat cultivars are susceptible to FGSC, the application of chemical fungicides has been a principal tool for controlling FHB in the last 40 decades. Previous studies have reported that the resistance of carbendazim is already widespread in China [8,9,30,31,32,33,34,35]. In addition, carbendazim can stimulate DON biosynthesis of FGSC, and carbendazim resistance can cause increase in DON production of FGSC [28,36]. A novel cyanoacrylate fungicide phenamacril exhibits a specific activity against *Fusarium* spp. and an inhibitory effect on DON production [10,26]. However, phenamacril has a high resistance risk in FGSC [10]. Previous studies have reported a strong correlation between FHB control efficacy and DON contamination [32,37,38,39]. Therefore, it is necessary to find novel fungicides for controlling FHB and DON contamination caused by FGSC.

Succinic dehydrogenase inhibitors (SDHIs) studied in this paper are respiratory inhibitors. They inhibit the transmission of electrons from succinic acid to ubiquinone by completely or partially occupying the ubiquinone site of the substrate, thus hindering the energy metabolism of bacteria, inhibiting the growth of pathogens, and achieving the purpose of controlling diseases [12]. Currently, only one SDHI fungicide carboxin has been registered for the control of FHB in China. Previous studies reported that SDHIs have good effects in the prevention and control of other diseases. For example, pydiflumetofen can effectively inhibit *Sclerotinia sclerotiorum*, with an average EC_50_ value of 0.0250 μg/mL [18]. In addition, boscalid and isopyrazam have a better inhibitory effect on mycelial growth of *Aspergillus flavus* than *Fusarium* species. Meanwhile, boscalid can reduce the toxin contamination of *A. flavus* [40].

In China, FGSC mainly includes *F. asiaticum* and *F. graminearum*. In this study, the sensitivity of seven *F. asiaticum* and six *F. graminearum* strains to five SDHIs (fluopyram, flutolanil, boscalid, benzovindiflupyr, and fluxapyroxad) was determined. We found that the five SDHIs did not differ in inhibiting the mycelial growth of FGSC, except for fluopyram. However, these five SDHIs exhibited a higher activity in inhibiting spore germination than mycelial growth. The results showed that the five SDHIs have potential in controlling FHB caused by FGSC in the field. Additionally, fluopyram exhibited a better inhibitory effect on either mycelial growth or spore germination in comparison to the other four SDHIs. In addition to fungicidal activity, we also found that these SDHIs can decrease DON production in *F. asiaticum* in vitro. At present, the biosynthetic pathway of DON has been extensively studied, and nearly all DON biosynthesis-involved genes (*TRI* genes) have been identified [21,41,42]. The trichothecene precursor synthase gene *TRI5* is a key enzyme in step one of DON biosynthesis [19]. In this study, five SDHIs caused a decrease in the *TRI5* gene expression. Moreover, we also observed that the five SDHIs could disrupt the formation of the complete spherical structure of toxisomes in *F. asiaticum*. The results revealed that the five SDHIs not only exhibited an inhibitory effect on spore germination in FGSC, but can also decrease DON biosynthesis in *F. asiaticum*. Thus, the five SDHIs have a potential in either controlling FHB or reducing DON contamination in *F. asiaticum*-infected grains.

The pyruvic acid produced in glycolysis is first transported into the mitochondria and oxidatively decarboxylated under aerobic conditions to form acetyl-CoA [43]. Meanwhile, acetyl-CoA is the major substrate for the biosynthesis of a variety of secondary metabolites, including trichothecenes [19]. Therefore, the content of pyruvic acid and acetyl-CoA in the strain should be positively correlated with the content of DON. Previous studies have shown that the production of pyruvic acid controlled by hexokinase supplies the main substrate for the biosynthesis of many secondary metabolites, such as trichothecene, DON, fumonisins, penicillin, and aflatoxin [20]. As expected, the content of pyruvic acid and acetyl-CoA were significantly reduced as affected by the five SDHIs. Pyruvic acid is the final product of glycolysis, and the decrease in its content indicated that the five SDHIs inhibited the glycolysis pathway. To verify this hypothesis, we performed qRT-PCR analysis of three key genes in the glycolysis pathway. We found that the relative expression of hexokinase and 6-phosphate fructokinase significantly decreased, and the relative expression of pyruvate kinase was significantly increased after treatment with the five SDHIs, resulting in inhibition of the glycolysis pathway. However, the relative expression of 6-phosphate fructokinase increased after treatment with benzovindiflupyr, possibly due to other action sites of benzovindiflupyr. The up-regulation of pyruvate kinase may be due to the product activation caused by the decrease of pyruvic acid content.

SDHIs affect the activity of succinate dehydrogenase, catalyzing the oxidation of succinic acid to fumaric acid in the TCA cycle. Isocitrate dehydrogenase is the rate-limiting enzyme of the TCA cycle [24,25]. Therefore, we determined the citric acid content, isocitrate dehydrogenase, and succinate dehydrogenase activities to investigate the regulatory effect of the five SDHIs in the TCA cycle. We found that the five SDHIs inhibited succinate dehydrogenase and isocitrate dehydrogenase activities, causing the decrease of the TCA cycle. In addition, the five SDHIs caused increase in citric acid content. Citric acid is the product of the first step of the TCA cycle [22], and its accumulation is due to the inhibition of downstream reactions. In addition, the accumulation of citric acid inhibits the glycolysis pathway [23]. SDHIs are respiratory inhibitors, which inhibit energy metabolism in bacteria [13,14]. Therefore, out of curiosity on whether SDHIs have the same effect on *F. asiaticum*, we determined the ATP content of *F. asiaticum* treated with five SDHIs. As expected, the ATP content was significantly reduced.

In summary, these five SDHIs exhibited inhibitory effects on the spore germination of FGSC. Importantly, SDHIs can decrease DON biosynthesis in *F. asiaticum* in vitro. This may be attributed to the inhibitory effects on glycolysis, TCA cycle, and energy metabolism caused by SDHIs. Thus, the results of the study will provide valuable information for wheat protection programs against the toxigenic fungi responsible for FHB and the consequent DON contamination in wheat grains.

## 4. Materials and Methods 

### 4.1. Fungicides, Fungal Strains and Culture Conditions

Technical-grade fluopyram, flutolanil, boscalid, benzovindiflupyr, and fluxapyroxad were kindly provided by Bayer (Shanghai, China), Nihon Nohyaku Co. (Shanghai, China), BASF (Shanghai, China), Syngenta (Beijing, China) and BASF (Shanghai, China), respectively. These fungicides were dissolved in methanol at 10 g/L and stored at 4 °C prior to further use.

Thirteen FGSC strains were isolated from the infected wheat ears in the field and stored in the Fungicide Biology Laboratory, Nanjing Agricultural University (Nanjing, China). These strains were identified by PCR assay as previously described [44]. The strain 2021, BM-1, BM-4, BM-13, BM-14, BM-17 and BM-20 are identified as *F. asiaticum*, and the strains BM-2, BM-3, BM-5, BM-7, BM-9 and BM-10 are identified as *F. graminearum*. *F. asiaticum* is dominant in eastern China, the most serious region affected by FHB. In addition, the strain 2021 was isolated from the infected wheat ear in 2000, and its genome was sequenced and analyzed in our laboratory. Thus, the strain 2021, as an *F. asiaticum* model strain, was selected for further research in this study.

Potato dextrose agar (PDA, 200 g/L potato, 20 g/L glucose and 20 g/L agar) was used for colony morphology examination and sensitivity test for five succinate dehydrogenase inhibitors in vitro. Water agar (WA, 16 g/L agar) was used for the determination of spore germination [45]. Mung bean broth (MBB, 30 g/L mung bean) was used for sporulation assays [46,47]. Yeast extract peptone dextrose medium (YEPD, 10 g/L peptone, 20 g/L glucose and 3 g/L yeast extract) was used for conidial germination. Glucose yeast extract peptone medium (GYEP, 1 g/L peptone, 50 g/L glucose and 1 g/L yeast extract) was employed for DON production. 

### 4.2. Fungicide Sensitivity Tests Based on Mycelial Growth and Spore Germination

Prior to fungicide sensitivity tests, the preliminary experiments were performed to optimize fungicide concentration gradients. For fungicide sensitivity tests, at least five concentrations for each fungicide were determined, and inhibition rate for all fungicide concentrations ranged 10% to 90%, and inhibition rate for medial concentration close to 50%. For mycelial growth, PDA plates were amended with fluopyram to obtain final concentrations of 2.5, 5, 10, 20, and 40 μg/mL and amended with the other four SDHIs (flutolanil, boscalid, benzovindiflupyr, and fluxapyroxad) to obtain final concentrations of 31.25, 62.5, 125, 250, and 500μg/mL, respectively. Inverted mycelial plugs (5 mm in diameter) cutting from the edge of an actively growing colony were transferred to 9 cm Petri dishes containing PDA media amended with the above described fungicide concentrations. Plates without fungicides were used as control. After incubation for 3 days in a growth chamber (25 °C), the colony diameters in two perpendicular directions for each PDA plate were measured and averaged. The EC_50_ values (effective concentration for 50% inhibition of mycelial growth) were calculated with the probit regression of the percentage of inhibition against the logarithmic value of fungicide concentrations.

For spore germination, WA plates were amended with fluopyram to obtain final concentrations of 0.125, 0.25, 0.5, 1, and 2 μg/mL and amended with the other four SDHIs (flutolanil, boscalid, benzovindiflupyr, and fluxapyroxad) to obtain final concentrations of 0.5, 1, 2, 4, and 8 μg/mL, respectively. Five mycelial plugs of each strain from the edge of 3-day-old colonies on PDA plates were transferred to a 50 mL flask containing 20 mL of mung bean broth. Conidia were filtered with two layers of lens wiping paper and collected by centrifuging at 5000 rpm for 5 min after culturing at 25 °C for 3 days in a shaker (175 rpm, 12 h of illumination every day). The conidia were suspended with sterile water, and the concentration was adjusted to 1 × 10^6^/mL. Then, 100 μL of conidia suspension was spread on WA plate containing the above described fungicide concentrations. After incubation for 5–6 h at 25 °C in the dark, the number of germinated conidia was measured. A conidium was considered germinated if the germ tube was at least half the length of the conidium. A total of 100 conidia were scored for each dish. The EC_50_ values (effective concentration for 50% inhibition of conidia germination) were estimated from the probit regression of the percentage of inhibition against the logarithmic value of fungicide concentrations [48]. Each concentration had three replicates, and the experiment was repeated twice.

### 4.3. RNA Extraction and Reverse Transcription PCR

The RNA simple Total RNA Kit (Tiangen, Beijing, China) was used to extract the total RNA from mycelia, and reverse transcription PCR was performed with the HiScript II qRT SuperMix for qPCR (+gDNA wiper) (Vazyme, Nanjing, China) as previously described [49]. The RNA integrity was validated by agarose gel electrophoresis and absorbance determination.

### 4.4. DON Production and TRI5 Gene Expression

For DON production, spore suspensions of the strain 2021 were prepared and diluted to 5 × 10^4^/mL. 1 mL of spore suspensions were added into 100mL GYEP [50,51]. After culturing in the dark at 28 °C for 24 h, the five SDHIs were added into the cultures and the final concentrations were 0.54 μg/mL for fluopyram, 2.96 μg/mL for flutolanil, 1.52 μg/mL for boscalid, 1.79 μg/mL for benzovindiflupyr, and 3.16 μg/mL for fluxapyroxad, respectively (the EC_50_ values are from the spore germination inhibition method and are listed in Table 2). After the incubation of an additive for 6 days, the culture liquid was collected and the mycelia were dried and weighed. DON production in the culture liquid was measured using the DON ELISA Kit (Wise, Zhenjiang, China) according to a previous study [7]. The DON ELISA Kit uses an indirect competitive ELISA method to detect the DON content in strains and cereals. Compared with the previous HPLC method, it is faster and easier to operate, while it also has higher detection accuracy. Its detection range is from 10 μg/L to 135 μg/L. DON production ability in shake culture was expressed as the amount of DON produced per dry weight of mycelia (μg/g). The experiment was repeated three times independently, with each treatment having three replicates.

For *TRI5* gene expression, the conidia of 2021 were added to GYEP (5 × 10^4^ conidia per 100 mL GYEP). After culturing for 24 h at 28 °C in the dark, the concentrations of five SDHIs were then added to the cultures as described above. After the incubation of an additive for 2 days, mycelia were collected for extraction of total RNA as previously described. *TRI5* gene expression was determined by qRT-PCR using the primers listed in Table 3, as described in Section 4.3. The experiment was repeated twice, with each treatment having three replicates.

### 4.5. Expression of Key Genes in Glycolysis Pathway

To determine the expression levels of key genes in glycolysis, the conidia of 2021 were added to YEPD (1 × 10^5^ conidia per 100 mL YEPD). After culturing for 24 h at 25 °C, the concentrations of five SDHIs were added to the cultures as described above. After the incubation of an additive for 2 days, mycelia were collected for extraction of total RNA as previously described. The expression levels of key genes in glycolysis were determined by qRT-PCR with the primers listed in Table 3. All data were normalized to actin gene expression, and relative changes in gene expression levels were analyzed with the CFX Manager Software (3.1, Bio-Rad, Hercules, CA, USA), which automatically sets the baseline. The experiments were repeated three times, with each treatment having three replicates.

### 4.6. Determination of Pyruvic acid, Acetyl-CoA, ATP and Citric Acid

The conidial suspensions were added to YEPD (1 × 10^5^ conidia per 100mL YEPD) and incubated for 24 h, then the concentrations of five SDHIs were added to the cultures as described above. After incubation with an additive for 2 days, the mycelia were collected and used for the determination of pyruvic acid, acetyl-CoA, ATP, and citric acid. Pyruvic acid and acetyl-CoA were assayed using a pyruvic acid content test kit (Solarbio, BC2205, Beijing, China) and an acetyl-CoA content test kit (Solarbio, BC0980, Beijing, China), respectively. ATP and citric acid production were assayed using an ATP assay kit (Beyotime, S0026, Nanjing, China) and a citric acid content test kit (Solarbio, BC2150, Beijing, China), respectively. In short, 0.05g of mycelia were added to the corresponding lysis buffer of different detection kits. After the lysis of mycelia, pyruvic acid, acetyl-CoA, ATP, or citric acid production were determined according to the manufacturer’s instructions. The experiments were performed three times independently.

### 4.7. SDH, ICDHm and NADH Dehydrogenase Activities

The mycelia were collected as described in Section 4.6 and used for the activities of SDH and ICDHm. SDH and ICDHm activities were determined using a succinate dehydrogenase activity assay kit (Solarbio, BC0950, Beijing, China) and an isocitrate dehydrogenase mitochondrial activity assay kit (Solarbio, BC2160, Beijing, China), respectively. The experiments were performed three times independently, with each treatment having three replicates.

NADH dehydrogenase activity was defined as the rate of decomposition of NADH. This experiment reflects the change of NADH by measuring the change of absorbance at 340 nm [52,53]. The mycelia were collected as described in Section 4.6. The mycelia (0.05 g) were ground with 1 mL of PBS phosphate buffer. The extracts were centrifuged for 10 min at 10,000 rpm at 4 °C, and the supernatant was ultrasonically broken. Finally, 100 μL of NADH (40 μmol) was added to 100 μL supernatant, and the changes in absorbance at 340 nm for 15 min were recorded using a spectrophotometer. The experiments were performed three times independently, with each treatment having three replicates.

### 4.8. Microscopic Examinations

In order to observe the morphological changes of the toxisomes, the strain 2021-*TRI1*-GFP labeled with *TRI1*-GFP was cultured in GYEP at 28 °C for 24 h, then the different concentrations of five SDHIs were added to the cultures as described above and cultured at 28 °C for 48 h. All samples were mounted on glass slides and sealed with cover glasses. Images of toxisomes were obtained at room temperature using a LEICA TCS SP8 confocal laser-scanning microscope (LEICA, laser: At 488 nm). The experiment was performed three times independently.

### 4.9. Statistical Analysis

All the data in this study were analyzed with the SPSS 14.0 software (SPSS Inc. Chicago, IL, USA) to obtain statistical variances between repeated experiments. Fisher’s LSD test (*p* = 0.05) was used to obtain the standard errors and determine whether there were significant differences among the biological characteristics.

## Figures and Tables

**Table 1:** Sensitivity of *Fusarium graminearum* species complex (FGSC) to five succinate dehydrogenase inhibitors (SDHIs) fungicides based on mycelial growth.

Fungicide	EC_50_ (μg/mL) ^a^												
	2021	BM-1	BM-4	BM-13	BM-14	BM-17	BM-20	BM-2	BM-3	BM-5	BM-7	BM-9	BM-10
Fluopyram	10.00 ± 1.22	9.29 ± 0.32	8.83 ± 0.35	1.65 ± 0.17	9.68 ± 0.40	7.79 ± 0.41	2.29 ± 0.26	6.65 ± 0.38	2.25 ± 0.31	9.65 ± 0.02	5.19 ± 0.23	6.32 ± 0.30	7.54 ± 0.20
Flutolanil	>100	>100	>100	>100	>100	>100	>100	>100	>100	>100	>100	>100	>100
Boscalid	>100	>100	>100	>100	>100	>100	>100	>100	>100	>100	>100	>100	>100
Benzovindiflupyr	>100	>100	>100	>100	>100	>100	>100	>100	>100	>100	>100	>100	>100
Fluxapyroxad	>100	>100	>100	>100	>100	>100	>100	>100	>100	>100	>100	>100	>100

^a^ Values are the means ± SEs of three replicate experiments.

**Table 2:** Sensitivity of FGSC to five SDHIs fungicides based on spore germination.

Fungicide	EC_50_ (μg/mL) ^a^												
	2021	BM-1	BM-4	BM-13	BM-14	BM-17	BM-20	BM-2	BM-3	BM-5	BM-7	BM-9	BM-10
Fluopyram	0.54 ± 0.11	0.74 ± 0.08	0.66 ± 0.08	0.39 ± 0.05	0.67 ± 0.06	0.56 ± 0.04	0.56 ± 0.06	0.54 ± 0.03	0.52 ± 0.05	0.68 ± 0.08	0.54 ± 0.05	0.69 ± 0.14	0.61 ± 0.03
Flutolanil	2.96 ± 0.21	3.23 ± 0.06	2.68 ± 0.07	2.47 ± 0.04	3.52 ± 0.10	3.01 ± 0.04	2.33 ± 0.03	2.84 ± 0.01	2.78 ± 0.01	4.24 ± 0.05	2.50 ± 0.07	2.99 ± 0.07	2.32 ± 0.04
Boscalid	1.52 ± 0.11	1.78 ± 0.08	2.58 ± 0.07	1.19 ± 0.05	2.11 ± 0.04	2.09 ± 0.03	1.57 ± 0.07	2.07 ± 0.03	1.28 ± 0.09	2.41 ± 0.10	3.06 ± 0.06	2.37 ± 0.06	2.40 ± 0.05
Benzovindiflupyr	1.79 ± 0.08	2.86 ± 0.09	2.43 ± 0.06	1.96 ± 0.04	2.02 ± 0.07	2.39 ± 0.05	2.20 ± 0.03	2.59 ± 0.04	2.03 ± 0.03	2.98 ± 0.08	2.15 ± 0.03	2.92 ± 0.06	2.74 ± 0.06
Fluxapyroxad	3.16 ± 0.05	2.95 ± 0.05	3.00 ± 0.04	2.30 ± 0.05	3.72 ± 0.06	3.99 ± 0.04	2.08 ± 0.04	3.19 ± 0.05	3.35 ± 0.06	3.78 ± 0.06	3.36 ± 0.06	3.15 ± 0.02	3.27 ± 0.05

^a^ Values are the means ± SEs of three replicate experiments.

**Table 3:** Primers used in this study.

Primer	Sequence (5′-3′)	Use
FGSG_*TRI5*-qF	CACTTGTCAAGGAGCACTTTC	qRT-PCR for determining the expression of *TRI5* (FGSG_03537)
FGSG_*TRI5*-qR	TGCTCAATCCAACATCCCTC	
Actin-qF	ATCCACGTCACCACTTTCAA	qRT-PCR for inter-reference actin
Actin-qR	TGCTTGGAGATCCACATTTG	
FGSG_00500-qF	CGACCTCCACGACAACA	qRT-PCR for determining the expression of hexokinase (FGSG_00500)
FGSG_00500-qR	GATAGCAGCAACGCCACA	
FGSG_09456-qF	GATAGATTGGAGAGCCGAGAGA	qRT-PCR for determining the expression of 6-phosphate fructokinase (FGSG_09456)
FGSG_09456-qR	GAGGTGCTGGATACACTTGATG	
FGSG_07528-qF	GAGATCCGAACTGGTAAGACTC	qRT-PCR for determining the expression of pyruvate kinase (FGSG_07528)
FGSG_07528-qR	CGTCAGAAGCGGTAGCATAA	
*TRI1*-GFP-F	ACTCACTATAGGGCGAATTGGGTACTCAAATTGGTTTTGTGAGTAGGCCTCATA	A pair of PCR primers to amplify *TRI1*(FGSG_00071) fragments used for construction of the *TRI1*-GFP, vector under its own promoter
*TRI1*-GFP-R	CACCACCCCGGTGAACAGCTCCTCGCCCTTGCTCACGTCATCCTGTACCAATTCCAATCG	
